# Diode Laser and Radiofrequency for Genitourinary Syndrome of Menopause: A Comparative Analysis

**DOI:** 10.3390/healthcare14050554

**Published:** 2026-02-24

**Authors:** Mariachiara Palucci, Marta Barba, Alice Cola, Yoav Baruch, Desirèe De Vicari, Matteo Frigerio

**Affiliations:** 1 Department of Gynecology, Fondazione IRCCS San Gerardo dei Tintori, University of Milano-Bicocca, 20900 Monza, Italy; m.barba8792@gmail.com (M.B.); alice.cola1@gmail.com (A.C.); d.devicari@campus.unimib.it (D.D.V.); frigerio86@gmail.com (M.F.); 2 Urogynecology and Pelvic Floor Unit, Lis Hospital for Women’s Health, Sourasky Medical Center, Gray Faculty of Medical & Health Sciences, Tel Aviv University, Tel Aviv-Yafo 6997801, Israel; yoavi100@gmail.com

**Keywords:** genitourinary syndrome of menopause (GSM), vulvovaginal atrophy (VVA), radiofrequency (RF), lasers, energy-based device (EBD), sexual dysfunction

## Abstract

**Background/Objectives**: Genitourinary syndrome of menopause (GSM) is a chronic, progressive condition that deeply affects sexual wellbeing and vaginal health. As many women—especially cancer survivors—seek non-hormonal alternatives, energy-based devices have gained increasing interest. However, comparative data between different technologies remain limited. This retrospective, non-randomized study aimed to directly compare the effectiveness and tolerability of fractional diode laser and monopolar radiofrequency (RF) in women with GSM. **Methods:** The study included 91 women treated with diode laser (*n* = 43) or RF (*n* = 48). Baseline evaluation comprised the Female Sexual Function Index (FSFI-19), Vaginal Health Index (VHI), and symptom severity. Post-treatment assessment included FSFI-19, VHI, Patient Global Impression of Improvement (PGI-I), and procedural discomfort (VAS 0–100). **Results:** Both modalities resulted in clear clinical benefits. Among women treated with the diode laser, FSFI total scores rose from 11.0 ± 8.4 to 15.3 ± 9.8 (*p* < 0.001), while VHI improved from 12.6 ± 3.0 to 15.9 ± 3.6 (*p* < 0.001). Similarly, RF treatment increased FSFI scores from 8.9 ± 7.4 to 14.3 ± 9.5 (*p* < 0.001) and VHI from 13.5 ± 3.0 to 16.5 ± 3.3 (*p* < 0.001). The overall degree of improvement was comparable between groups (ΔFSFI: 4.3 ± 6.5 vs. 5.4 ± 7.1; ΔVHI: 3.3 ± 2.9 vs. 3.0 ± 3.0). Despite this, a higher proportion of patients in the RF group reported PGI-I scores < 4 (95.5% vs. 74.3%; *p* = 0.010), in parallel with significantly lower procedural discomfort compared to laser treatment (VAS 14.1 vs. 53.6; *p* = 0.001). No adverse events were observed. **Conclusions:** Vaginal diode laser and monopolar RF proved to be effective, non-hormonal interventions capable of improving sexual function and restoring vaginal health in women with GSM. However, RF demonstrated superior tolerability, suggesting it may provide a more comfortable therapeutic experience without sacrificing clinical effectiveness.

## 1. Introduction

Genitourinary Syndrome of Menopause (GSM) is a prevalent chronic condition affecting a large proportion of postmenopausal women, which encompasses a spectrum of vulvovaginal and lower urinary tract symptoms, including vaginal dryness, burning, irritation, dyspareunia, urinary urgency, and recurrent infections [1]. The pathophysiology of GSM is primarily related to estrogen deficiency, which leads to epithelial thinning, decreased vascularization, loss of elasticity, reduced collagen content, and an altered vaginal microbiota and pH. These changes contribute to significant discomfort, impaired sexual function, and a decline in overall quality of life [2,3].

GSM is estimated to affect around 50% of postmenopausal women [4]. With the continuing rise in average life expectancy, many women now spend more than one-third of their lives in menopause [5]. Despite its high prevalence and significant impact on quality of life (QoL), GSM remains underdiagnosed and undertreated. This is largely due to the common perception that GSM symptoms are a natural part of aging and something women must simply accept, as well as limited awareness among healthcare providers about the condition’s prevalence and clinical manifestations [6].

Multiple treatment modalities are available for GSM, aimed at mitigating symptoms of vulvovaginal atrophy and promoting the restoration of urogenital physiology. Current evidence supports a stepwise, individualized approach to treatment, beginning with non-hormonal interventions such as vaginal moisturizers, lubricants, and lifestyle measures aimed at reducing irritative factors [7]. For patients with persistent or moderate-to-severe symptoms, low-dose vaginal estrogen therapy remains the gold standard due to its efficacy in restoring epithelial integrity, improving vaginal pH, and relieving dyspareunia while maintaining minimal systemic absorption [8]. Alternative hormonal options include intravaginal dehydroepiandrosterone (prasterone) [9] and selective estrogen receptor modulators such as ospemifene [10], which provide symptom relief for women who cannot or prefer not to use local estrogen. Despite the availability of different therapeutic strategies, many of these are not recommended for specific subgroups, particularly breast cancer survivors, due to the lack of long-term safety data and concerns regarding the potential risk of recurrence [11,12]. Overall, in breast and gynecologic cancer survivors with symptoms unresponsive to non-hormonal measures, any low-dose vaginal hormones may be considered with shared decision-making in conjunction with patients’ oncologists [13,14]. In this regard, contraindications, safety concerns and poor adherence to hormonal therapies underscore the need for alternative therapeutic strategies.

Over the past decade, energy-based devices (EBDs)—including fractional microablative CO_2_ lasers, non-ablative erbium:YAG lasers and diode lasers, and radiofrequency platforms—have emerged as innovative, minimally invasive modalities aimed at restoring vaginal tissue integrity through controlled thermal stimulation and neocollagenesis [15]. Although EBDs share the ultimate goal of enhancing vaginal tissue function, their underlying mechanisms of action differ significantly. In detail, fractional CO_2_ and Er:YAG lasers rely on selective photothermal absorption by water, generating either microablative injury or controlled non-ablative heating that activates fibroblasts, improves extracellular matrix turnover, and promotes epithelial thickening [15,16,17]. In contrast, diode lasers, typically functioning within the 1470–1550 nm range, induce homogeneous, non-ablative thermal diffusion into the deeper lamina propria, stimulating synthesis of type I and III collagen, improving vascular perfusion, and increasing tissue elasticity without causing epithelial disruption [18,19]. Radiofrequency (RF) devices differ even more fundamentally, as they do not operate through light–tissue interaction but instead generate endogenous heating through high-frequency electromagnetic currents that induce ionic agitation and resistive heating. This mechanism results in immediate collagen fiber contraction, progressive neocollagenesis, and enhanced blood flow, all while avoiding tissue ablation, which supports their favorable safety profile [20,21]. These divergent thermal behaviors and tissue penetration depths are clinically significant, as they influence patient selection, treatment protocols and expected outcomes.

Despite the growing number of studies reporting symptomatic improvement and histological enhancement following the use of EBDs for GSM, these technologies have not yet been incorporated into major clinical guidelines. This gap is primarily attributable to the heterogeneity of existing studies, the predominance of small, uncontrolled or observational designs, and the limited availability of long-term safety and efficacy data [22,23,24]. Moreover, variability in treatment protocols, device parameters, and patient selection criteria complicates direct comparison across studies and limits the generalizability of findings. In light of these uncertainties, our study aims to contribute meaningful evidence by providing a comparative analysis of our clinical experience with diode laser therapy and radiofrequency treatment, two non-ablative modalities with distinct mechanisms of action but shared therapeutic goals. By evaluating their relative effectiveness, safety, and patient-reported outcomes within a consistent clinical framework, we seek to clarify their role and potential positioning in the future management of GSM.

## 2. Materials and Methods

This retrospective, comparative, non-randomized study was conducted at the Department of Obstetrics and Gynecology of Fondazione IRCCS San Gerardo dei Tintori, a tertiary-care university hospital in Northern Italy. The study analyzed medical records of women treated between September 2022 and June 2025 for symptoms related to GSM using either diode laser or RF therapy. All data were extracted from the hospital’s electronic health archive. Both treatments under investigation were approved by the Institutional Ethics Committee of our hospital (protocol codes: GSM-LASER and GSM-RF), and written informed consent was obtained from all participants before the procedures were performed. Patients who underwent one of the two treatments—diode laser or radiofrequency—were considered eligible only if they met all of the following criteria: they had experienced at least two GSM-related symptoms for a minimum of one year, were aged ≥ 18 years, and had contraindications to local estrogen therapies or declined their use. All consecutive patients meeting the inclusion criteria during the study period were included. Patients were allocated to the diode laser group or the RF group based on the treatment received as part of routine clinical care and on device availability over time: patients treated between 2022 and 2023 received diode laser therapy, whereas patients treated from late 2024 through 2025 received RF following the introduction of this technology at our institution.

The laser treatment consisted of three-monthly sessions performed with the Leonardo Dual diode laser (Biolitec Italia Srl, Milano, Italy), a dual-wavelength device (980 nm and 1470 nm) designed to achieve different tissue interactions. Each session was carried out according to the manufacturer’s protocol, using a power setting of 7 W in pulsed mode (two 0.5 s pulses separated by a 0.5 s pause). After applying a local lidocaine gel, a vaginal glass handpiece was introduced into the vaginal canal, followed by insertion of the dedicated optical fiber. The procedure was performed following the producer’s recommendations, delivering eight circumferential pulses per centimeter of vaginal length, from the fornix to the introitus. While, the radiofrequency treatment consisted of a cycle of five weekly sessions, each lasting 20 min. A monopolar capacitive intravaginal radiofrequency device C500 Urogyne (Capenergy, Medswiss, Barcelona, Spain) was used, equipped with an active intracavitary electrode inserted into the vagina and a dispersive passive electrode positioned over the lumbosacral region. Before application, the active probe was covered with a non-lubricated condom and a water-based gel was applied to facilitate smooth movement over the vulvar area and comfortable insertion into the vaginal canal. Power and frequency settings were adjusted to maintain temperatures below 39 °C externally and below 42 °C during intravaginal delivery, in accordance with safety recommendations.

At the first and final treatment sessions, patients were clinically evaluated using several standardized tools. The Vaginal Health Index (VHI) was used to assess vaginal trophism, comprising five parameters: elasticity, fluid volume, pH, epithelial integrity, and moisture. The total score ranges from 5 to 25, with scores below 15 indicating the presence of atrophic vaginitis [25]. In addition, sexual function was assessed using the Female Sexual Function Index (FSFI-19), a 19-item self-administered questionnaire that evaluates six domains: sexual desire, arousal, lubrication, orgasm, satisfaction, and pain. Each item is rated on a 5-point Likert scale, with a total score ranging from 2 to 36 [26]. Finally, the Patient Global Impression of Improvement (PGI-I) and procedural discomfort, measured using a 0–100 Visual Analog Scale (VAS) [27], were assessed at the end of the treatment sessions. All patients were closely monitored throughout the treatment period for the occurrence of any adverse effects.

Continuous variables were reported as mean ± standard deviation (SD), while categorical variables were expressed as frequencies and percentages. The assumption of approximate normality was evaluated by visual inspection of data distributions. In light of the retrospective study design, potential heterogeneity of variances, and unequal group sizes, Welch’s *t*-test was consistently applied for all between-group comparisons of continuous variables, given its robustness to violations of homoscedasticity and normality assumptions. Baseline comparability between groups was assessed using Welch’s *t*-test for continuous variables and Chi-square tests for categorical variables, including oncologic history, previous pelvic surgery, and parity. Treatment effectiveness was evaluated by calculating Δ values for each outcome measure, defined as the difference between post-treatment and pre-treatment scores (post − pre). These Δ values, reflecting the magnitude of clinical improvement, were compared between groups using Welch’s *t*-test. Within-group changes over time were analyzed using paired *t*-tests.

All statistical analyses were performed using IBM SPSS Statistics for Windows, Version 29.0 (IBM Corp., Armonk, NY, USA) [28]. A *p*-value < 0.05 was considered statistically significant.

## 3. Results

A total of 91 women were included in the study: 43 treated with diode laser and 48 with radiofrequency (RF). The two cohorts showed similar demographic and clinical profiles at baseline as shown in Table 1. Women in the laser group had a mean age of 56.6 years, whereas those in the RF group had a mean age of 53.9 years, with no statistically significant difference between groups. The distribution of oncologic patients was also comparable (51.2% in the laser group and 58.3% in the RF group), as was the prevalence of previous pelvic surgery (60.5% vs. 54.2%). Parity rates were balanced, with approximately two-thirds of patients in both groups having had at least one childbirth. These findings indicate that the two populations were broadly comparable at baseline.

Baseline functional assessments confirmed that both groups presented with significant sexual dysfunction. The mean FSFI total score was low in both cohorts (11.2 in the laser group and 8.7 in the RF group), without statistically significant differences. All FSFI domains—desire, arousal, lubrication, orgasm, satisfaction, and pain—were similarly compromised in the two groups. Baseline VHI values also showed no substantial differences between the cohorts, confirming similar degrees of vulvovaginal atrophy and dryness symptoms prior to treatment. The baseline clinical characteristics of the two study populations are summarized in Table 2.

**Figure 1:**
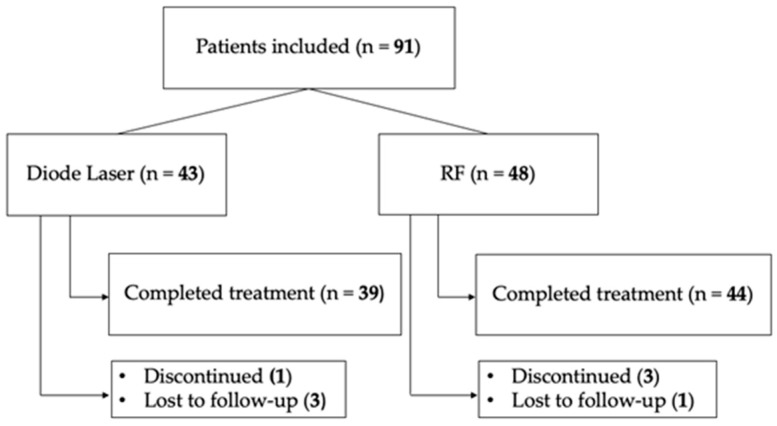
Study flow chart illustrating patient inclusion, treatment allocation, and follow-up in the diode laser and radiofrequency (RF) groups.

A small number of participants did not complete the treatment or the post-treatment assessment as graphically shown in the flow chart in Figure 1. In the Laser group, three women were lost to follow-up and one discontinued treatment due to comorbidities, resulting in four non-completers. In the RF group, three women discontinued the treatment sessions prematurely and one was lost to follow-up, for a total of four non-completers. Therefore, all pre–post and Δ (post–pre) analyses were conducted on 39 completers in the Laser group and 44 completers in the RF group, while all baseline analyses included the full initial sample.

Regarding treatment efficacy, both interventions were associated with clinically meaningful and statistically significant improvements in sexual function and vaginal health as described in Table 3. In the laser group, all FSFI domains showed significant pre–post increases, with improvements ranging from approximately 1.5 to 3 points depending on the domain. The FSFI total score increased by more than 4 points on average from 11.0 to 15.3 and the VHI also showed a marked and statistically significant improvement (12.6 to 15.9 *p* < 0.001), indicating enhanced vaginal health after treatment. A similar pattern was observed in the RF group. All FSFI domains and the FSFI total score improved significantly following treatment, often with magnitudes comparable to those observed after laser therapy. The VHI also increased significantly (13.5 to 16.5, *p* < 0.001), confirming improvement in vulvovaginal symptoms. No adverse events were reported by any patient in either treatment group. Within each group, these ameliorations of symptoms were confirmed by paired statistical analyses.

When directly comparing the magnitude of improvement between the two treatments, no statistically significant differences were found for any FSFI domain, FSFI total score, or VHI. Although RF showed slightly larger numerical gains in several FSFI domains, and laser showed marginally greater improvement in VHI, none of these differences reached statistical significance (*p* > 0.05). These findings indicate that the two modalities offer comparable clinical effectiveness, with neither showing superiority over the other based on the analyzed outcomes, as comprehensively detailed in Table 4.

**Figure 2:**
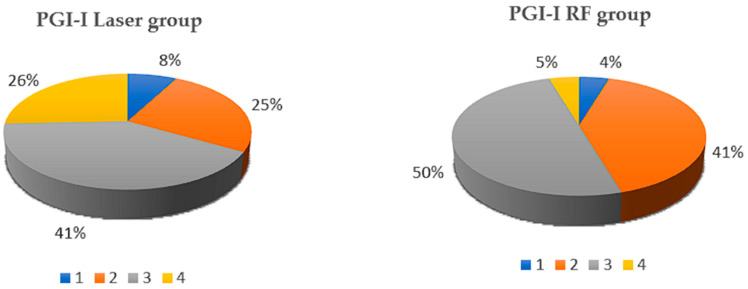
PGI-I score distribution in the Laser and RF groups. 1 = Very much improved, 2 = Much improved, 3 = Minimally improved, 4 = No change.

Patient-reported procedural experience highlighted notable differences between the two treatments as highlighted in Table 5 and in Figure 2. Perceived benefit, defined as PGI-I score < 4, was also substantial, but significantly higher for RF compared to laser (95.5% vs. 74.3%; *p* = 0.010). Furthermore, procedural discomfort as measured by VAS differed markedly (53.6 vs. 14.1, *p* < 0.001), with laser treatment associated with significantly higher scores. These findings suggest that RF may offer superior procedural tolerability despite comparable clinical effectiveness.

Overall, diode laser and radiofrequency were equally effective in enhancing sexual function and vaginal health, showing similarly robust clinical improvements. The distinction between the two modalities was driven more by procedural tolerability than by differences in therapeutic benefit.

## 4. Discussion

Genitourinary syndrome of menopause (GSM), of which vulvovaginal atrophy (VVA) represents a core component, remains a highly prevalent yet frequently underrecognized condition among postmenopausal women [29]. Despite their substantial impact on sexual function, urinary comfort, and overall quality of life, these conditions are often underdiagnosed or misattributed to natural aging or to consequences of oncological treatments [30,31]. This diagnostic gap may arise from both patients’ reluctance to report intimate symptoms and limited clinician awareness of the chronic, progressive nature of estrogen deprivation-related vulvovaginal changes [32].

Local estrogen therapy remains the gold standard for treating vulvovaginal atrophy, as it directly compensates for hypoestrogenism and has been shown to restore epithelial thickness, increase glycogen content, improve vascularity, and enhance collagen and elastin structure. Through these mechanisms, estrogen effectively reverses many of the histological alteration characteristics of GSM [33,34]. However, despite their proven efficacy, vaginal estrogens are contraindicated or used with extreme caution in breast cancer survivors, particularly those with estrogen receptor–positive disease or those receiving aromatase inhibitors, due to persistent concerns about systemic absorption and potential stimulation of residual malignant cells [13,35,36]. These safety concerns also extend to many women in the general population, who frequently discontinue or avoid hormone-based treatments because of the perception—often exaggerated—of an increased risk of developing malignancies [34,37]. As a result, a substantial proportion of symptomatic women remain undertreated, highlighting the urgent need for effective, non-hormonal alternatives capable of restoring vaginal health without posing oncologic risks.

In this scenario, energy-based devices have emerged as promising minimally invasive treatments for all those women who have no benefits, contraindications, or are not compliant with the other available first-line or hormonal therapies. Among these, fractional micro-ablative CO_2_ lasers were the first to be widely studied and demonstrated significant improvements in sexual function, vaginal health, and symptoms such as dyspareunia and dryness [38,39]. More recently, non-ablative technologies (non-ablative Er:YAG lasers, radiofrequency, and diode lasers) have gained attention as safer alternatives. By delivering controlled thermal stimulation without epithelial damage, they promote collagen remodeling, improved vascularization, and enhanced vaginal hydration and elasticity, with less discomfort and downtime [40,41].

Despite growing evidence supporting both laser and RF technologies, direct comparative studies remain scarce, with most trials being single-arm designs or involving heterogeneous treatment settings. This underscores the relevance of the present study, which provides a direct, head-to-head comparison between vaginal diode laser and monopolar RF. Both treatments proved effective in improving sexual function and vaginal health, with significant pre- to post-treatment gains in FSFI score and VHI in each group (*p* < 0.001). When the magnitude of improvement was compared between modalities, however, no significant difference emerged. The ΔFSFI (4.3 vs. 5.4) and ΔVHI (3.3 vs. 3.0) were comparable, indicating that neither treatment demonstrated superior clinical efficacy.

Importantly, although post-treatment FSFI-19 values remained below levels consistent with full recovery of sexual function, the observed improvements represent a clinically meaningful benefit in a population characterized by severe baseline impairment and limited therapeutic options.

What did differ was procedural tolerability: RF was associated with markedly lower procedural discomfort (VAS 14.1 vs. 53.6; *p* < 0.001), suggesting a more favorable treatment experience. This finding is supported by the routine application of a lidocaine-based anesthetic before diode laser procedures, highlighting the attention required to ensure adequate procedural tolerability. As PGI-I is a subjective global outcome measure, differences in procedural comfort may have influenced PGI-I ratings, partially explaining the higher proportion of RF patients reporting scores < 4 (*p* = 0.010), despite similar objective treatment effects. An important aspect to consider when interpreting patient-reported outcomes is the difference in treatment schedules between the two modalities. The radiofrequency protocol involved more frequent but shorter weekly sessions, whereas diode laser treatment was delivered in monthly sessions. Differences in treatment frequency, cumulative exposure, and overall treatment burden may have influenced procedural discomfort and global perception of improvement, independently of the intrinsic efficacy of the devices.

From a clinical perspective, the comparable effectiveness observed between diode laser and radiofrequency suggests that treatment selection may reasonably be guided by factors such as procedural tolerability, patient preference, treatment schedule, and resource availability rather than expected differences in efficacy. The lower discomfort associated with radiofrequency may represent a meaningful advantage in terms of patient acceptance and satisfaction, particularly in populations requiring repeated or maintenance treatments.

In terms of cost-effectiveness perspective, the two technologies also differ in terms of acquisition, maintenance, and operational requirements. Diode laser systems generally entail a higher initial investment and maintenance burden and require the use of a reusable glass vaginal handpiece, which must be sterilized after each treatment session, with additional logistical and processing considerations. By contrast, radiofrequency devices are typically associated with lower acquisition costs and simpler operational workflows. Although a formal cost-effectiveness analysis was beyond the scope of the present study, these differences may be relevant in real-world clinical settings, influencing resource allocation, workflow efficiency, and accessibility of treatment.

Consistent with the existing literature, the device-specific outcomes observed in our study align with previously published clinical investigations. For example, Gambacciani et al. reported a clear improvement in FSFI scores after three sessions of a 1470 nm diode laser, together with significant reductions in vaginal dryness and dyspareunia (*p* < 0.001) [42]. Similar findings were described by Perrini et al., who observed that a course of dual-wavelength diode laser therapy resulted in marked clinical benefits, including a reduction in vulvovaginal symptom severity, an 8.8-point increase in the total FSFI score and a 7.5-point improvement in the Vaginal Health Index, while maintaining an excellent safety profile with no serious adverse events [43].

Evidence supporting radiofrequency therapies is equally encouraging. In a prospective study, Sekiguchi et al. reported significant improvements in vaginal laxity and sexual function following a full cycle of monopolar radiofrequency treatments, with FSFI scores increasing from approximately 22 to 26 (*p* = 0.002) [44]. These findings were reinforced by Millheiser et al., who showed that even a single monopolar RF session produced clinically meaningful benefits, including an increase in Vaginal Laxity Questionnaire (VLQ) scores from 2.5 to 4.0 (*p* < 0.001), improved sexual satisfaction, and high patient satisfaction rates, with clinical effects persisting for up to six months [21]. Overall, both radiofrequency and dual-wavelength diode laser therapies appear to achieve clinical improvements comparable to those reported for other non-ablative energy-based devices, particularly non-ablative Er:YAG lasers. In a recent sham-controlled study, Bayraktar et al. demonstrated that non-ablative Er:YAG laser therapy led to significant improvements in both objective and subjective GSM parameters, including an approximately 4-point increase in VHI, significant reductions in dyspareunia, and measurable gains in vaginal wall elasticity assessed by shear-wave elastography [45].

However, as previously introduced, a consistent limitation in the current literature is the absence of rigorous comparative studies evaluating the relative performance of different energy-based technologies—including radiofrequency, diode lasers, Er:YAG, and CO_2_ devices—in the treatment of GSM. Most available evidence examines each modality in isolation, typically through small prospective cohorts or observational studies lacking standardized protocols [16,46]. As highlighted by Benini et al., research in this field suffers from substantial heterogeneity in laser parameters, session number, outcome measures, and follow-up duration, which makes cross-technology comparisons scientifically unreliable [47]. In the same vein, more recent analyses reiterate these concerns, indicating that the absence of adequately powered head-to-head trials and the reliance on subjective endpoints leave the evidence base fragmented and insufficient for definitive comparative assessment of available technologies [48,49]. However, one pattern appears relatively consistent: ablative devices—particularly fractional CO_2_ lasers—are more often associated with transient adverse effects such as burning sensations, mucosal irritation, and mild edema, whereas such reactions are reported less frequently with radiofrequency and other non-ablative modalities [49,50]. Taken together, the literature suggests that although multiple energy-based technologies demonstrate therapeutic benefit in GSM, radiofrequency tends to offer a more favorable tolerability profile without sacrificing clinical effectiveness, a trend that aligns with the findings of our study.

To the best of our knowledge, this is one of the first comparative studies evaluating fractional diode laser and monopolar radiofrequency. Despite its retrospective design, the study benefits from a well-defined dataset with consistent clinical documentation and standardized outcome measures, including FSFI-19, its individual domains, VHI, PGI-I, and procedural VAS. These validated tools allowed for a comprehensive assessment of both subjective symptoms and objective vaginal health. Baseline comparability between groups was confirmed through appropriate statistical testing (Welch’s *t*-test and Chi-square), strengthening the reliability of the comparative results. Importantly, no adverse events were reported in either group, further supporting the safety of both treatments in routine clinical practice.

However, several limitations should be considered. First, the retrospective and non-randomized design inherently limits causal inference. Although baseline characteristics were comparable between groups, treatment allocation reflected routine clinical practice and device availability over time rather than predefined clinical criteria; therefore, unmeasured factors such as symptom severity, patient expectations, or clinician preference may have influenced treatment allocation, and potential selection bias cannot be excluded. Second, the limited sample size, although adequate for detecting within-group changes, may be underpowered to identify small differences in efficacy between treatments. Third, the study relied on short-term follow-up, preventing conclusions about long-term durability of the effects, which would be an interesting aspect to explore in future research. In addition, treatment protocols were based on standard clinical practice, but energy settings, operator experience, or anatomical variability might introduce performance heterogeneity. Lastly, participants were drawn from a single clinical center, which could limit the generalizability of the results to broader populations.

## 5. Conclusions

Radiofrequency and diode laser both represent effective and minimally invasive outpatient options for the management of GSM, offering meaningful improvements in vaginal function, sexual wellbeing, and mucosal health. Their safety and tolerability profiles make them particularly attractive for women who cannot use, do not respond to, or prefer to avoid local estrogen therapy. In this comparative analysis, the two modalities showed comparable short-term therapeutic benefits according to QoL scales. Notably, RF was associated with significantly better procedural tolerability and higher patient-reported global improvement. Although objective efficacy measures were similar between groups, these differences in treatment experience may represent a clinically meaningful advantage in terms of patient acceptance and overall treatment satisfaction in real-world practice. Future research should focus on prospective, adequately powered comparative studies with longer follow-up to assess durability of benefits, optimal treatment scheduling, and maintenance strategies. Further efforts are also needed to identify patient characteristics that may predict differential response to specific energy-based modalities, thereby supporting more personalized management of GSM. Such evidence will help position these technologies more accurately within the evolving therapeutic framework for GSM.

## Figures and Tables

**Table 1:** Baseline population characteristics. Continuous variables are reported as mean ± standard deviation (SD), and categorical variables as absolute and relative frequencies. *p*-values were calculated using Welch’s *t*-test for continuous variables and Chi-square tests for categorical variables.

Population Characteristics	Laser Group	RF Group	*p*-Value
Age (Years)	56.6 ± 9.9	53.9 ± 10.0	0.236
Parous women (%)	26 (60.5%)	27 (56.3%)	0.246
Previous pelvic surgery (%)	26 (60.5%)	26 (54.2%)	0.694
Oncological history (%)	22 (51.2%)	28 (58.3%)	0.635

**Table 2:** Baseline values of sexual function (FSFI total score and individual FSFI domains) and vaginal health (VHI), expressed as mean ± standard deviation (SD) for both treatment groups. *p*-values refer to Chi-square tests for categorical variables and Welch’s *t*-test for continuous variables.

	Laser Group	RF Group	*p*-Value
Total FSFI-19 score	11.2 ± 8.4	8.7 ± 7.2	0.139
Desire	3.7 ± 1.9	3.1 ± 1.2	0.116
Arousal	6.2 ± 5.7	4.5 ± 4.6	0.173
Lubrication	5.5 ± 5.8	4.2 ± 5.4	0.295
Orgasm	4.2 ± 4.5	3.3 ± 3.9	0.349
Satisfaction	5.8 ± 4.1	4.2 ± 3.5	0.081
Pain	3.5 ± 4.4	2.9 ± 3.5	0.492
VHI	12.9 ± 3.2	13.5 ± 3.0	0.402
Dropouts	4 (9.3%)	4 (8.3%)	1.000

**Table 3:** Pre- and post-treatment comparisons for each treatment group (Laser and RF). Data are reported as mean ± standard deviation (SD). FSFI-19: Female Sexual Function Index; VHI: Vaginal Health Index.

	Laser PRE	Laser POST	Laser *p*	RF PRE	RF POST	RF *p*
Total FSFI-19 score	11.0 ± 8.4	15.3 ± 9.8	*p* < 0.001	8.9 ± 7.4	14.3 ± 9.5	*p* < 0.001
Desire	3.8 ± 1.9	4.8 ± 2.1	0.001	3.2 ± 1.3	4.5 ± 1.8	*p* < 0.001
Arousal	6.3 ± 5.8	9.0 ± 5.9	0.003	4.5 ± 4.8	7.3 ± 5.4	*p* < 0.001
Lubrication	5.6 ± 5.7	8.3 ± 6.4	0.002	4.3 ± 5.6	7.4 ± 6.5	*p* < 0.001
Orgasm	4.3 ± 4.5	6.1 ± 5.3	0.005	3.5 ± 4.0	5.8 ± 5.1	*p* < 0.001
Satisfaction	5.6 ± 4.1	7.9 ± 5.1	0.001	4.4 ± 3.6	7.1 ± 4.6	*p* < 0.001
Pain	3.5 ± 4.1	5.0 ± 5.2	0.016	3.0 ± 3.6	5.2 ± 5.1	0.002
VHI	12.6 ± 3.0	15.9 ± 3.6	*p* < 0.001	13.5 ± 3.0	16.5 ± 3.3	*p* < 0.001

**Table 4:** Comparison of Δ (post–pre) scores for FSFI total, FSFI domains, and VHI between the Laser and RF groups. Values are expressed as mean ± SD. *p*-values refer to Welch’s *t*-test comparing changes between groups.

	Δ Laser	Δ RF	*p*-Value
Total FSFI-19 score	4.3 ± 6.5	5.4 ± 7.1	0.474
Desire	1.1 ± 1.8	1.3 ± 1.6	0.521
Arousal	2.7 ± 5.4	2.8 ± 4.2	0.960
Lubrication	2.7 ± 5.2	3.1 ± 5.1	0.743
Orgasm	1.8 ± 3.7	2.3 ± 3.8	0.511
Satisfaction	2.3 ± 3.8	2.7 ± 3.6	0.650
Pain	1.5 ± 3.7	2.1 ± 4.3	0.457
VHI total	3.3 ± 2.9	3.0 ± 3.0	0.608

**Table 5:** Comparison PGI-I scores and procedural VAS between Laser and RF groups. Continuous data are reported as mean (SD), and non-continuous data are reported as absolute (relative) frequency. PGI-I (Patients Global Impression of Improvement); VAS (Visual Analog Scale).

	Laser Group	RF Group	*p*-Value
PGI-I < 4	29/39 (74.4%)	42/44 (95.5%)	0.010
Procedural VAS	53.6 ± 13.7	14.1 ± 9.7	*p* < 0.001

## Data Availability

The data presented in this study are available upon request from the corresponding author due to privacy and ethical considerations.

## Abbreviations

The following abbreviations are used in this manuscript:

GSM	Genitourinary Syndrome of Menopause
QoL	Quality of life
EBD	Energy-based device
FSFI-19/FSFI	Female Sexual Function Index
VHI	Vaginal Health Index
PGI-I	Patient Global Impression of Improvement
VAS	Visual Analog Scale
RF	Radiofrequency
CO_2_	Carbon Dioxide (laser)
DHEA	Dehydroepiandrosterone
SD	Standard Deviation
SPSS	Statistical Package for the Social Sciences
IBM	International Business Machines
VLQ	Vaginal Laxity Questionnaire
VVA	Vulvovaginal Atrophy
Er:YAG (o YAG)	Erbium:Yttrium-Aluminum-Garnet (laser)
